# Global Prevalence and Associated Factors of Medication Errors in Hospitalized Pediatric Patients: Systematic Review and Meta-Analysis

**DOI:** 10.2196/100070

**Published:** 2026-08-27

**Authors:** Xiuwen Chen, Yuan Meng, Xueyi Wei, Ailian Li, Jiqun He, Liqing Yue

**Affiliations:** 1 Xiangya School of Nursing, Central South University, Changsha, Hunan, China; 2 National Clinical Research Center for Geriatric Disorders, Xiangya Hospital Central South University, Changsha, Hunan, China; 3 Xiangya Research Center of Evidence-based Healthcare, Central South University, Changsha, Hunan, China; 4 Teaching and Research Section of Clinical Nursing, Xiangya Hospital Central South University, No. 87 Xiangya Road, Kaifu District, Changsha, Hunan, 410028, China, 86 073184327394

**Keywords:** medication errors, pediatric patients, hospitalized children, meta-analysis, prevalence, associated factors, systematic review

## Abstract

**Background:**

Medication errors represent a major patient safety concern among hospitalized children due to developmental variability, weight-based dosing, and limited communication capacity. However, the global burden and determinants of pediatric inpatient medication errors remain insufficiently characterized.

**Objective:**

This study aims to estimate the worldwide prevalence of medication errors in hospitalized pediatric patients and to identify factors associated with their occurrence.

**Methods:**

A systematic review and meta-analysis was conducted using Embase, MEDLINE, Web of Science, PubMed, and the Cochrane Library from database inception to June 2025. Observational studies involving pediatric inpatients younger than 18 years were included. The review followed the Preferred Reporting Items for Systematic Reviews and Meta-Analyses (PRISMA) 2020 statement. Data were pooled using a DerSimonian-Laird random-effects model, with subgroup and sensitivity analyses performed to explore heterogeneity.

**Results:**

A total of 61 studies from 29 countries or regions were included. The pooled prescribing error rate was 28%, the pooled administration error rate was 32%, and 54% of the hospitalized pediatric patients experienced at least one medication error. Prescribing error rates were higher in lower-middle–income countries (44%), intensive care units (34%), antibiotic prescriptions (30%), and studies conducted between 2013 and 2025 (32%). The proportion of patients experiencing medication errors was the highest in upper-middle–income countries (64%), whereas administration error rates were the highest in low-income countries (71%), although these subgroup findings should be interpreted cautiously because of limited numbers of studies and wide CIs. Factors associated with medication errors included intravenous administration (odds ratio [OR] 6.86, 95% CI 3.17‐14.83), hospital stay longer than 5 days (OR 1.94, 95% CI 1.33‐2.81), and prescription of 3 or more medications (OR 2.12, 95% CI 1.66‐2.70).

**Conclusions:**

Medication errors are highly prevalent among hospitalized children worldwide, with substantial variation by region, income level, care setting, and time period. Errors are closely associated with treatment-related factors, underscoring the need for targeted system-level strategies to improve pediatric medication safety.

## Introduction

Medication errors have become a major public health issue threatening patient safety worldwide [1] and are particularly prominent in pediatric health care settings [2]. Children and neonates are reported to be up to 3 times more likely to experience medication errors than adults [3,4]. Due to the limited availability of pediatric-specific formulations, adult medications often require dose calculations, manipulation, or reconstitution to meet individualized pediatric treatment needs [5,6]. In addition, factors such as complex dose calculations [7], age-related differences in drug metabolism [8], the use of unlicensed or off-label medications [8], and children’s limited ability to communicate symptoms and treatment-related concerns [9] may further increase the risk of medication errors. These errors may lead to adverse drug events, prolonged hospitalization, increased health care costs, and even irreversible harm or death [10]. Medication errors impose a substantial burden on health care systems worldwide. In the United States, medication errors are associated with annual costs exceeding US $29 billion, including an estimated additional US $28 million in hospital costs each year [11,12]. In the United Kingdom, medication errors result in economic losses of £9,846,258 annually for the National Health Service and consume a considerable number of bed-days [13]. The World Health Organization has estimated that medication errors cost approximately US $42 billion globally each year [14].

The National Coordinating Council for Medication Error Reporting and Prevention (NCC MERP) [15] defines a medication error as any preventable event that may cause or lead to inappropriate medication use or patient harm while the medication is in the control of a health care professional, patient, or consumer. These events may be related to professional practice, health care products, procedures, or systems and may involve prescribing, order communication, product labeling, packaging, nomenclature, compounding, dispensing, administration, education, monitoring, and use. Medication errors thus span multiple stages of the medication-use process. Prior studies [16] suggest that most medication errors in children occur during the prescription, dispensing, and administration stages. Among them, prescription errors account for 3% to 37% of children’s medication errors, dispensing errors account for 5% to 58%, and administration errors account for 72% to 75% of children’s medication errors [17].

Although numerous studies in recent years have examined the epidemiology of pediatric medication errors [18-20], substantial methodological heterogeneity persists. Inconsistencies in the operational definitions of medication errors, variation in data collection methods, diversity of study settings, and differences in denominators have resulted in wide variability in reported prevalence, limiting comparability across studies. Gates et al [21] stratified pooled estimates of medication error prevalence in hospitalized children by ward type and use of health information technology, but their work focused solely on quantitative synthesis of prevalence without systematically addressing associated factors. This lack of root cause analysis limits the ability to develop efficient and targeted prevention strategies in clinical practice.

To address these gaps, we conducted a systematic review and meta-analysis with two primary objectives: (1) to evaluate the occurrence of medication errors among hospitalized pediatric patients worldwide and (2) to identify factors associated with medication errors in hospitalized children. By consolidating epidemiological evidence, our study aims to support the development of more precise and evidence-based strategies for the prevention of pediatric inpatient medication errors.

## Methods

### Search Strategy and Study Selection

The systematic review and meta-analysis was conducted and reported in accordance with the Preferred Reporting Items for Systematic Reviews and Meta-Analyses (PRISMA) 2020 statement (Checklist 1). A comprehensive search was conducted in Embase, Web of Science, the Cochrane Library, MEDLINE, and PubMed from database inception to June 2025. A combination of MeSH terms and free-text terms was used, including “children,” “hospitalized,” and “medication errors.” Detailed search strategies for each database are provided in Multimedia Appendix 1. This study was registered with PROSPERO (registration number: CRD42024613159).

Inclusion criteria were (1) hospitalized pediatric patients aged less than 18 years, including those in general pediatric wards, pediatric intensive care units (PICUs), and neonatal intensive care units (NICUs); (2) studies reporting the prevalence of medication errors or investigating associated factors; and (3) observational studies (cross-sectional, cohort, or case-control).

Exclusion criteria were (1) studies focusing exclusively on specific patient populations (eg, oncology patients) or specific drug categories; (2) studies based solely on voluntary reporting systems, as these systems rely on active reporting by health care professionals and may underestimate the true prevalence of medication errors; however, studies that combined voluntary reporting with active surveillance methods, such as direct observation, medical record review, or other systematic detection approaches, were included; (3) non-English publications; and (4) conference abstracts, posters, studies with insufficient data, or studies for which the full text was unavailable.

### Data Extraction and Quality Assessment

Two investigators independently extracted data using Microsoft Excel, including first author, publication year, country or region, study design, ward type, study period, age range or mean age (SD) of included children, data collection methods, source of medication error definition, prevalence of medication errors (%), and associated factors. Discrepancies were resolved by a third investigator. For overlapping datasets, the study with the larger sample size was prioritized.

Cohort and case-control studies were assessed using the Newcastle-Ottawa Scale (NOS), with scores of 0 to 3, 4 to 6, and 7 to 9 indicating low, moderate, and high quality, respectively [22]. Cross-sectional studies were evaluated using the Agency for Healthcare Research and Quality (AHRQ) criteria, with scores of 0 to 3, 4 to 7, and 8 to 11 indicating low, moderate, and high quality, respectively [23]. Studies of all quality levels were included to capture all available evidence in the field.

### Outcome Definitions

This study initially planned to extract data on various types of medication errors, including prescribing errors, dispensing errors, and administration errors. However, because some error types were reported in only a limited number of studies and exhibited substantial variability in reporting methods, meta-analyses were conducted only for outcomes that met the criteria for quantitative synthesis, namely the proportion of patients experiencing medication errors, prescribing error rates, and administration error rates.

To avoid misinterpretation arising from differences in units of analysis, the outcome measures were defined as follows. The prescribing error rate was defined as the proportion of prescriptions containing at least one medication error among all prescriptions, reflecting the occurrence of medication errors during the prescribing process. The administration error rate was defined as the proportion of medication administrations involving at least 1 medication error among all medication administrations, reflecting the occurrence of medication errors during the medication administration process. The proportion of patients experiencing medication errors was defined as the proportion of patients who experienced at least one medication error during the observation period among all patients, reflecting the overall burden of medication errors among hospitalized pediatric patients. Because these outcomes were calculated using different units of analysis (prescriptions, medication administrations, and patients), their estimates represent different aspects of medication error occurrence and should not be directly compared.

### Statistical Analysis

All statistical analyses were performed using Stata (version 17.0). Neither the Freeman-Tukey double arcsine transformation nor the logit transformation was applied to the original proportions. For the meta-analysis of medication error occurrence among hospitalized pediatric patients, the effect size was the medication error rate. For the meta-analysis of factors associated with medication errors, the effect size was expressed as the odds ratio (OR) with its corresponding 95% CI. Given the substantial heterogeneity among the included studies (*I*²> 50% and *P*<.05), pooled estimates were calculated using the DerSimonian-Laird random-effects model. Subgroup analyses were conducted according to relevant study characteristics and associated factors to explore potential sources of heterogeneity. To assess the robustness of the findings, several sensitivity analyses were performed. A leave-one-out analysis was conducted to evaluate the influence of individual studies on the pooled estimates. In addition, restricted maximum likelihood (REML) estimation was applied within the random-effects framework to examine the impact of different τ² estimation methods on the pooled results. Publication bias was assessed using both Egger’s test and visual inspection of funnel plot symmetry. All statistical tests were 2-sided, and *P*<.05 was considered statistically significant.

## Results

### Literature Search

**Figure 1.**
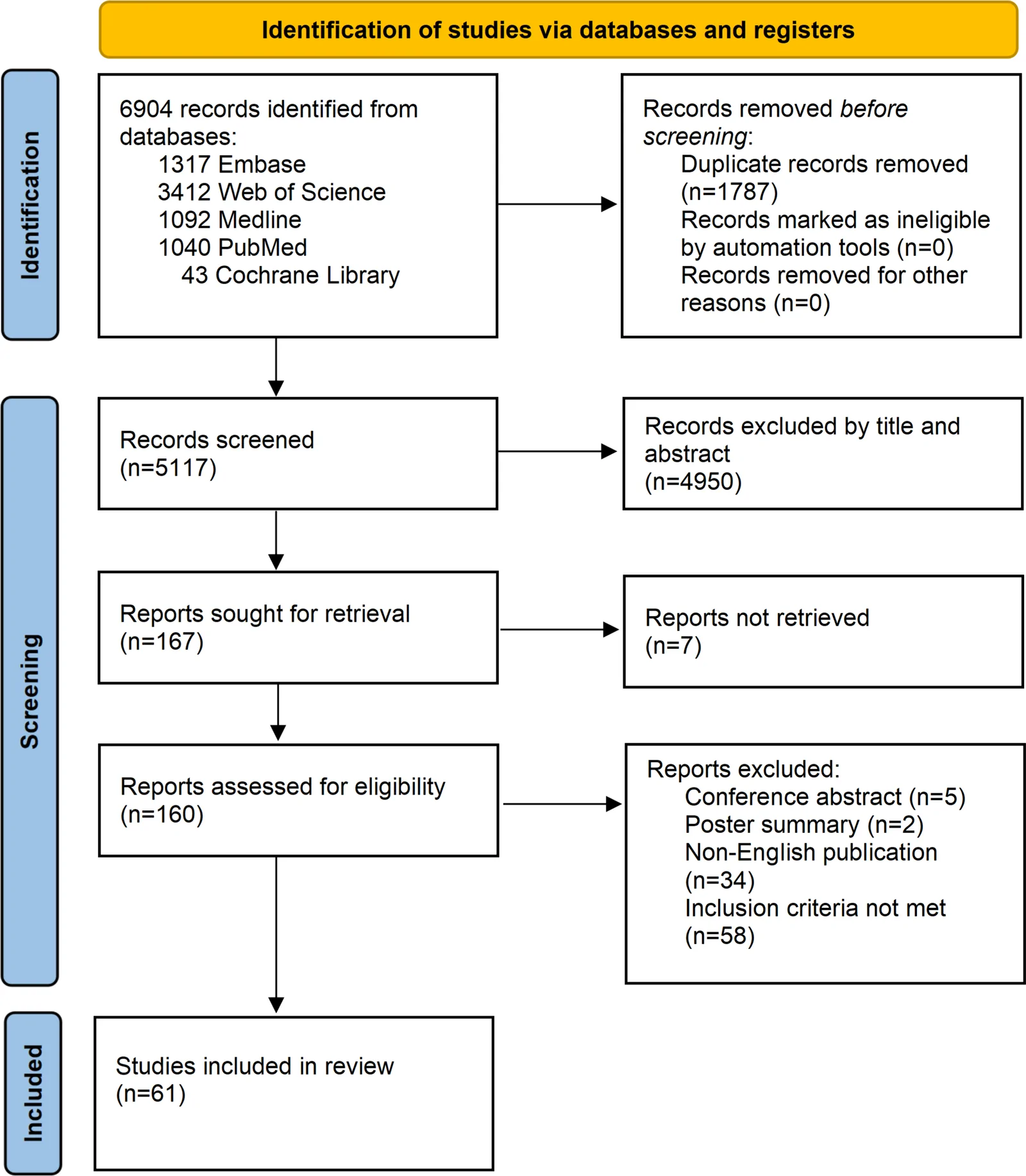
Flow diagram of the study selection process.

A total of 6904 records were identified through searches of Embase, Web of Science, the Cochrane Library, MEDLINE, and PubMed. After removing 1787 duplicates, 4950 records were excluded based on titles and abstracts, and 7 reports were excluded because the full text was not available. Following full-text review, 99 articles were further excluded for not meeting the inclusion criteria, leaving 61 studies for analysis. The study selection process is summarized in Figure 1.

### Characteristics and Quality Assessment of the Included Studies

Details of the included studies are provided in Table 1, with sample sizes ranging from 20 to 8691 participants. The studies were conducted across 29 countries or regions, including 14 high-income, 8 upper-middle–income, 4 lower-middle–income, and 3 low-income countries. Ward types included general pediatric wards, PICUs, and NICUs. Among the included studies, 35 were cross-sectional, with AHRQ scores ranging from 4 to 7 (Table S1 in Multimedia Appendix 2), and 26 were cohort studies, with NOS scores ranging from 3 to 9 (Table S2 in Multimedia Appendix 2). To ensure that all available data were used to estimate medication error rates, no studies were excluded based on methodological quality; all original studies reporting medication errors were included.

The types of medication errors reported in the included studies included prescribing errors, administration errors, dispensing errors, and medication reconstitution errors. However, because only a limited number of studies reported on dispensing errors and medication reconstitution errors, and substantial variability existed in outcome definitions and reporting methods, reliable quantitative synthesis was not feasible for these error types. Therefore, meta-analyses were conducted only for the proportion of patients experiencing medication errors, prescribing error rates, and administration error rates among hospitalized pediatric patients.

**Table 1.** Basic characteristics of included studies.

First author (year)	Country or region	Study design	Ward type	Study period	Age range or mean (SD)^a^	Data collection method	Source of medication error definition	Medication error rate	Associated factors
Canales-Siguero et al [24], 2025	Spain	Cross-sectional	NICU^b^	2021‐2022	34 weeks (median, IQR 30‐39)	Medical record review	Reference	Prescription errors: 3.3%	Birth weight, gestational age, ward occupancy
Henry Basil et al [25], 2025	Malaysia	Cohort	NICU	2022‐2023	35 weeks (median, IQR 30‐39)	Direct observation	Reference	Administration errors: 68.0%; proportion of patients experiencing medication errors: 92.4%	Medications administered intravenously, unavailability of a protocol, the number of prescribed medications, nursing experience, nonventilated neonates, and gestational age in weeks
Badgery-Parker et al [26], 2024	Australia	Cohort	Medical, surgical, and day-stay wards	2016‐2020	0‐18 years (range^c^)	Direct observation; medical record review	NR^d^	Prescription errors: 18.6%; administration errors: 36.2%	Age
Westbrook et al [27], 2024	Australia	Cohort	General pediatric, medical, and surgical wards	22 weeks	0‐16 years (range)	Direct observation; medical record review	Reference	Administration errors: 37.0%	Intravenous route, early morning and weekend administrations, patient age ≥11 years, oral medications requiring solvents or diluents, and eMM^e^ use
Abiri et al [28], 2024	Sierra Leone	Cross-sectional	Pediatric ward	2021	0‐18 years (range)	Medical record review	NCC MERP^f^	Proportion of patients experiencing medication errors: 56.1%	NR
Kwaku Wuni et al [29], 2024	Ghana	Cross-sectional	Pediatric and the newborn care units	2022	1.37 years (mean, SD 0.83)	Questionnaire survey	Reference	Proportion of patients experiencing medication errors: 65.9%	Ward type
Satir et al [30], 2023	Switzerland	Cohort	Pediatric ward	2018‐2019	0‐18 years (range)	Medical record review	Reference	Prescription errors: 49.55%	NR
Bante et al [31], 2023	Ethiopia	Cross-sectional	Pediatric ward	2020‐2021	≤28 days to ≥10 years (range)	Questionnaire survey	NCC MERP	Proportion of patients experiencing medication errors: 69.5%	Work environment, child weight, education level of medication provider, parental involvement, adherence to administration authority, length of hospital stay
Xavier et al [32], 2022	Mozambique	Cross-sectional	Pediatric ward	2020	0‐10 years (range)	Medical record review	WHO^g^, BNFC^h^, NICE^i^	Prescription errors: 34.9%; proportion of patients experiencing medication errors: 36.5%	Number of prescribed medications, length of hospital stay
Akkawi et al [33], 2022	Malaysia	Cross-sectional	Pediatric ward	2019	0‐12 years (range)	Medical record review	National Antibiotic Guideline	Proportion of patients experiencing medication errors: 57.9%; prescription errors: 27.9%	Infection type, number of antibiotics used
Garedow et al [34], 2022	Ethiopia	Cross-sectional	Pediatric ward	2021	0‐18 years (range)	Medical record review	NR	Prescription errors: 19.29%	Length of hospital stay, number of prescribed medications, empirical therapy
Nasso et al [35], 2022	Italy	Cohort	Pediatrics, pediatric nephrology, and pediatric rheumatology	2019	0‐18 years (range)	Medical record review	WHO	Prescription errors: 56.0%	Ward type
Alekaw et al [36], 2022	Ethiopia	Cross-sectional	Pediatric ward	2020‐2021	1.96 years (mean, SD 3.48)	Medical record review	NR	Proportion of patients experiencing medication errors: 30.8%	Age, parental negligence, residence
Özdemir et al [37], 2021	Turkey	Cross-sectional	Pediatric ward	2016	1-226 months (range)	Database; medical record review	Reference	Proportion of patients experiencing medication errors: 40.4%	NR
Abdel-Qader et al [38], 2021	Jordan	Cross-sectional	NICU, PICU, general pediatrics, surgical ICU, pediatric surgery	2019	2.1 years (mean)	Medical record review	NR	Prescription errors: 31.5%	Number of prescribed medications
Kadmon et al [39], 2021	Israel	Cohort	PICU^j^	2015‐2016	6.5 years (mean)	Database	NR	Prescription errors: 1.6%	Age, disease severity
Yehualaw et al [40], 2021	Ethiopia	Cross-sectional	Pediatric ward	2018‐2019	3.26 years (median, IQR 2‐4)	Medical record review	NR	Proportion of patients experiencing medication errors: 28.3%	Age
Bharathi et al [41], 2020	India	Cohort	NICU	2016	NR	Voluntary reporting; interview; medical record review	Reference	Proportion of patients experiencing medication errors: 22%	Polypharmacy, length of hospital stay
Brennan-Bourdon et al [42], 2020	México	Cross-sectional	PEC^k^, NICU, NIMCU, PICU	2017‐2018	0‐18 years (range)	Medical record review	NCC MERP	Prescription errors: 53.5%	NR
Howlett et al [43], 2020	Ireland	Cross-sectional	PICU	2017	0‐16 years (range)	Bedside direct observation; electronic prescription system	NCC MERP	Administration errors: 13.0%	NR
McMullan et al [44], 2020	Australia	Cross-sectional	Pediatric ward	2014‐2017	0‐18 years (range)	Standardized online audit tool	NR	Prescription errors: 19.6%	Admission to nontertiary pediatric hospital, hospital location in nonmajor city
Bonafide et al [45], 2020	United States	Cohort	PICU	2016‐2017	<6 months to ≥18 years (range)	Telecommunications and electronic health record data	Investigator-defined	Administration errors: 3.2%	Mobile phone interruptions, nursing experience, nurse-to-patient ratio, patient care level
Ramírez-Camacho et al [46], 2020	México	Cross-sectional	NICU	NR	NR	Direct observation	NCC MERP	Administration errors: 34.6%	NR
Eslami et al [47], 2019	Iran	Cross-sectional	NICU	2016	NR	Medical record review	NCC MERP	Proportion of patients experiencing medication errors: 74.8%; prescription errors: 42.0%	Gestational age, length of hospital stay
Fekadu et al [48], 2019	Ethiopia	Cross-sectional	Pediatric ward	2017	1 month to 12 years (range)	Medical record and medication chart review	NR	Proportion of patients experiencing medication errors: 68.0%	Critical illness, administration route, number of prescribed medications
Palmero et al [49], 2019	Switzerland	Cross-sectional	NICU	2010-2012	33.4 weeks (mean)	Direct observation	NCC MERP	Patient-related errors: 84.8%	Number of prescribed medications, gestational age
Tang et al [50], 2018	Malaysia	Cross-sectional	Pediatric ward	NR	0.1-16.1 years (range)	Direct observation	ASHP^l^	Prescription errors: 1.5%; administration errors: 10.4%	Intravenous formulations, gastrointestinal medications
Kadam et al [51], 2018	India	Cross-sectional	NICU	2013	NR	Medical record review	ASHP	Prescription errors: 65.6%	NR
Ergül et al [52], 2018	Turkey	Cross-sectional	ICU, hematology, infectious diseases, pediatric ward	2017	10‐80 months (range)	Medical record review	NR	Proportion of patients experiencing medication errors: 33.8%	NR
Baraki et al [53], 2018	Ethiopia	Cross-sectional	Pediatric ward, PICU and NICU	2016‐2017	1 day to 14 years (range)	Structured questionnaire; observation	WHO and NCC MERP	Administration errors: 62.3%	Patient age, education level of administering health care provider, availability of pharmacy preparation room, number of prescribed medications, availability of medication administration guidelines
Rishoej et al [54], 2018	Denmark	Cross-sectional	NICU, PICU and pediatric ward	2016	0‐16 years (range)	Unobtrusive direct observation; on-site recording	NCC MERP	Administration errors: 8.0%	NR
Truter et al [55], 2017	South Africa	Cross-sectional	Neonatal and pediatric ward	6 months	1 month to 19 years (range)	Direct observation; medical record and prescription review; medication error checklist	NCC MERP	Proportion of patients experiencing medication errors: 78.0%	NR
Al-Ramahi et al [56], 2017	Palestine	Cohort	Pediatric ward	2014	1‐182 months (range)	Medical record review	Drug information handbook	Prescription errors: 22.4%; proportion of patients experiencing medication errors: 40.0%	Age, weight, number of medications, length of hospital stay
Mekory et al [57], 2017	Israel	Cross-sectional	Pediatric ward	2013‐2014	0‐18 years (range)	Medical record review	ASHP	Prescription errors: 6.5%; administration errors: 11.3%	NR
Nikhithasri et al [58], 2017	India	Cross-sectional	PICU and NICU	6 months	0‐18 years (range)	Records; direct communication	NCC MERP	Prescription errors: 56.1%	NR
Khoo et al [59], 2017	Malaysia	Cross-sectional	Pediatric ward, PICU and NICU	2015	0‐18 years (range)	Drug chart review	NR	Prescription errors: 9.2%	NR
Ewig et al [60], 2017	China	Cohort	PICU	2015	3.2 years (mean, SD 3.2)	Medical record review	Institute of Medicine	310 errors per 100 admissions	NR
Tribble et al [61], 2020	United States	Cross-sectional	Medical, surgical, NICU and nonneonatal intensive care	2016‐2017	0‐17 years (range)	Electronic medical record review; standardized assessment form	NR	Proportion of patients experiencing medication errors: 25.7%; prescription errors: 21.0%	Prescription drug category, indication
Dedefo et al [62], 2016	Ethiopia	Cohort	Pediatric ward	2014	≤28 days to ≤14 years (range)	Medical record review	ASHP	Proportion of patients experiencing medication errors: 75.1%; prescription errors: 46.0%	Length of hospital stay, number of prescribed medications
Palmero et al [63], 2016	Switzerland	Cohort	NICU	8 months	33.1 weeks (mean)	Review of medical orders	Reference	Prescription errors: 28.9%	NR
Machado et al [64], 2015	Brazil	Cross-sectional	NICU	2011	NR	Medical record review	ASHP	Prescription errors: 43.5	Gestational age, weight
Glanzmann et al [65], 2015	Switzerland	Cohort	PICU	2010	NR	Medical record review	Reference	Prescription errors: 13.4%	NR
Stultz et al [66], 2014	United States	Cohort	General ward	2011	0‐17.8 years (range)	Electronic medical record; pharmacy record review	Guidelines and formularies	Prescription errors: 0.54%	NR
Sakuma et al [67], 2014	Japan	Cohort	Pediatric general ward, NICU, PICU, ICU, and emergent care unit	2009	2 years (median, IQR 0‐7)	Medical record review	NR	69.5 errors per 100 admissions	NR
Chedoe et al [68], 2012	The Netherlands	Cohort	NICU	2006	≥25 weeks (range)	Direct observation	NR	Administration errors: 48.6%	NR
Booth et al [69], 2012	United Kingdom	Cohort	PICU	32 weeks	1.65 years (median, IQR 0.35‐6.10)	Prescription review	Reference	892 errors per 1000 occupied bed-days	NR
Al-Jeraisy et al [70], 2011	Saudi Arabia	Cohort	PICU	5 weeks	0‐14 years (range)	Medical record review	Reference	Prescription errors: 56.0%	NR
Kunac et al [71], 2010	New Zealand	Cohort	NICU, postnatal ward and pediatric ward	2002	NR	Medical record review; multidisciplinary meeting; parent interview; voluntary report	NCC MERP	NR	Length of hospital stay, number of prescribed medications, administration route
Feleke et al [72], 2010	Ethiopia	Cross-sectional	Pediatric ward	2009	0‐15 years (range)	Direct observation	Reference	Administration errors: 89.9%	NR
Chua et al [73], 2010	Malaysia	Cross-sectional	General pediatric ward and pediatric oncology ward	2004‐2005	0‐16 years (range)	Direct observation	Reference	Administration errors: 11.7%	Ward type
Ghaleb et al [74], 2010	United Kingdom	Cross-sectional	Surgical, medical, intensive care (PICU or NICU), adolescent units	2004‐2006	NR	Drug chart review; unobtrusive observation	Reference	Prescription errors: 13.2%; administration errors: 19.1%	NR
Ligi et al [75], 2008	France	Cohort	NICU	2005	NR	Anonymous voluntary reporting system; independent observer verification; standardized form recording	Reference	4.9 errors per 100 admissions	NR
Campino et al [76], 2008	Spain	Cohort	NICU	NR	NR	Medical record review	Reference	Prescription errors: 32.8%; transcription error rate: 20.5%	NR
Parihar et al [77], 2008	India	Cohort	NICU, PICU, pediatric ward, and rooming-in labor ward	2005	28 weeks to 18 years (range)	Medical record review; interview	Reference	Prescription errors: 24.3%	NR
Otero et al [78], 2008	Argentina	Cross-sectional	NICU, PICU, pediatric ward	2002	0‐18 years (range)	Medical record review	ASHP	Prescription errors: 11.4%	Night shift, age, year of resident or researcher, assistant administration
Buckley et al [79], 2007	United States	Cohort	PICU	2004	0‐18 years (range)	Direct observation	Reference	Prescription errors: 14.6%	NR
Wang et al [80], 2007	United States	Cross-sectional	NICU, PICU, pediatric ward	2002	NR	Medical record review; voluntary reporting	Reference	Prescription errors: 5.1%	NR
Walsh et al [81], 2006	United States	Cohort	NICU, PICU, pediatric ward	2002‐2003	NR	Medical record review; computer log analysis; standardized form recording	Reference	Prescription errors: 1.5%	NR
Prot et al [82], 2005	France	Cross-sectional	PICU, NICU, pediatric nephrology unit, and general pediatric unit	2002‐2003	NR	Direct observation	ASHP	Administration errors: 31.3%	Drug category, administration route, nurse type, number of patient management protocols
Potts et al [83], 2004	United States	Cohort	Pediatric critical care unit	2001‐2002	6.5 years (mean, SD 12.0)	Medical record review	Investigator-defined	Prescription errors: 39.1%	NR
Kaushal et al [3], 2001	United States	Cohort	Pediatric general medical wards, general surgical wards, PICU, and NICU	1999	NR	Clinical staff reports and review of prescription sheets, medication administration records, and patient charts	Reference	Prescription errors: 5.7%	NR

a The age values are reported as range, mean (SD), or median (IQR) as available.

b NICU: neonatal intensive care unit.

c range: minimum-maximum.

d NR: not reported.

e eMM: electronic medication management system.

f NCC MERP: National Coordinating Council for Medication Error Reporting and Prevention.

g WHO: World Health Organization.

h BNFC: British National Formulary for Children.

i NICE: National Institute for Health and Care Excellence.

j PICU: pediatric intensive care unit.

k PEC: pediatric emergency care.

l ASHP: American Society of Health-System Pharmacists.

### Meta-Analysis of Prescribing Errors in Hospitalized Pediatric Patients

**Figure 2.**
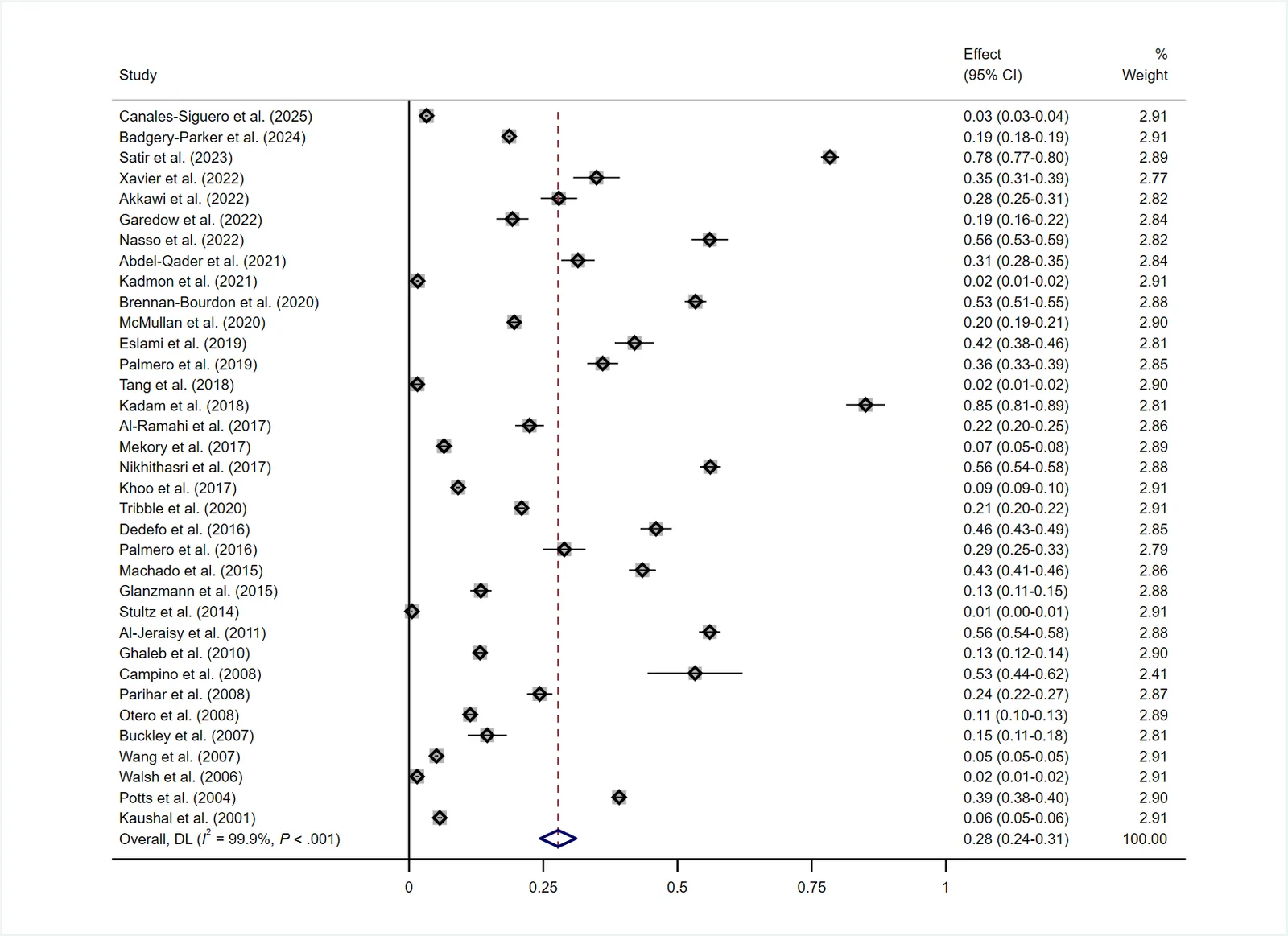
Forest plot of prescribing error rates in hospitalized pediatric patients. Weights are from random effects model. DL: DerSimonian-Laird [3,24,26,30,32-35,38,39,42,44,47,49-51,56-59,61-66,70,74,76-81,83].

Based on the denominators used in the included studies, we reported prescribing and administration error rates as well as the proportion of patients experiencing medication errors. Thirty-five studies [3,24,26,30,32-35,38,39,42,44,47,49-51,56-59,61-66,70,74,76-81,83] reported prescribing error rates in hospitalized children. Significant heterogeneity was observed among studies (*I*²=99%; *P<*.001). Sensitivity analyses using a leave-one-out approach indicated that no single study significantly influenced the overall results. Therefore, a random-effects model was applied. The pooled prescribing error rate among hospitalized pediatric patients was 28% (95% CI 24%‐31%; Figure 2).

### Subgroup Analysis of Prescribing Error Rates in Hospitalized Pediatric Patients

Subgroup analyses of prescribing error rates are presented in Table 2, with the number of included studies reported for each category. When stratified by study design, prescribing error rates were comparable between cohort studies (28%, 95% CI 23%‐34%) and cross-sectional studies (27%, 95% CI 22%‐32%). By country income level, prescribing error rates appeared higher in lower-middle–income countries (44%, 95% CI 24%‐64%) compared with low-income countries (33%, 95% CI 16%‐51%), although the number of studies in each subgroup was limited and CIs were wide. Regional subgroup analysis showed variability across geographic areas. The highest estimate was observed in Latin America and the Caribbean (36%, 95% CI 8%‐64%), whereas North America showed a lower estimate (12%, 95% CI 7%‐17%). However, these differences should be interpreted cautiously due to limited study numbers and substantial uncertainty. Regarding ward type, intensive care units showed higher prescribing error rates (34%, 95% CI 29%‐39%). For prescription types, antibiotic medications showed slightly higher error rates (30%, 95% CI 24%‐36%) compared with nonantibiotic medications (27%, 95% CI 24%‐31%). For temporal trends, studies conducted between 2013 and 2025 showed higher prescribing error rates (32%, 95% CI 26%‐38%) compared with those conducted between 2000 and 2012 (18%, 95% CI 14%‐22%). However, this finding should be interpreted cautiously given potential differences in study settings, definitions, and data collection methods across time periods.

**Table 2.** Subgroup analysis of prescribing error rates in hospitalized pediatric patients.

Subgroup	Studies, n	Events, n	Total, n	Prevalence (95% CI)	*P* value	*I*^2^ (%)
Study design						
Cross-sectional	19	13,398	89,380	0.27 (0.22-0.32)	<.001	99.85
Cohort	16	21,618	157,676	0.28 (0.23-0.34)	<.001	99.95
Country income level						
High income	20	27,301	206,580	0.23 (0.19-0.27)	<.001	99.95
Upper-middle income	7	4227	25,689	0.27 (0.16-0.38)	<.001	99.82
Lower-middle income	5	2489	5919	0.44 (0.24-0.64)	<.001	99.67
Low income	3	803	2244	0.33 (0.16-0.51)	NA^a^	NA
Study location						
Europe	8	3857	32,939	0.35 (0.15-0.55)	<.001	99.92
Asia	11	5897	28,962	0.31 (0.23-0.39)	<.001	99.89
Africa	3	803	2244	0.33 (0.16-0.51)	NA	NA
North America	7	8011	105,964	0.12 (0.07-0.17)	<.001	99.94
Latin America and the Caribbean	3	2101	5602	0.36 (0.08-0.64)	NA	NA
Australia and New Zealand	2	14,047	75,000	0.19 (0.18-0.19)	NA	NA
Special regions	1	213	949	0.22 (0.20-0.25)	NA	NA
Ward type						
Intensive care unit	17	10,070	89,161	0.34 (0.29-0.39)	<.001	99.91
General ward	11	17,588	83,764	0.30 (0.20-0.40)	<.001	99.87
Mixed wards	7	7358	74,131	0.10 (0.05-0.14)	<.001	99.84
Prescription type						
Antibiotic prescriptions	7	5971	26,589	0.30 (0.24-0.36)	<.001	98.81
Non-antibiotic prescriptions	28	29,045	220,467	0.27 (0.24-0.31)	<.001	99.94
Study period						
2000‐2012	11	6027	87,882	0.18 (0.14-0.22)	<.001	99.87
2013‐2025	18	25,435	142,088	0.32 (0.26-0.38)	<.001	99.92
NA	6	3554	17,086	0.33 (0.17-0.50)	<.001	99.90

a NA: not applicable.

### Meta-Analysis of the Proportion of Patients Experiencing Medication Errors

**Figure 3.**
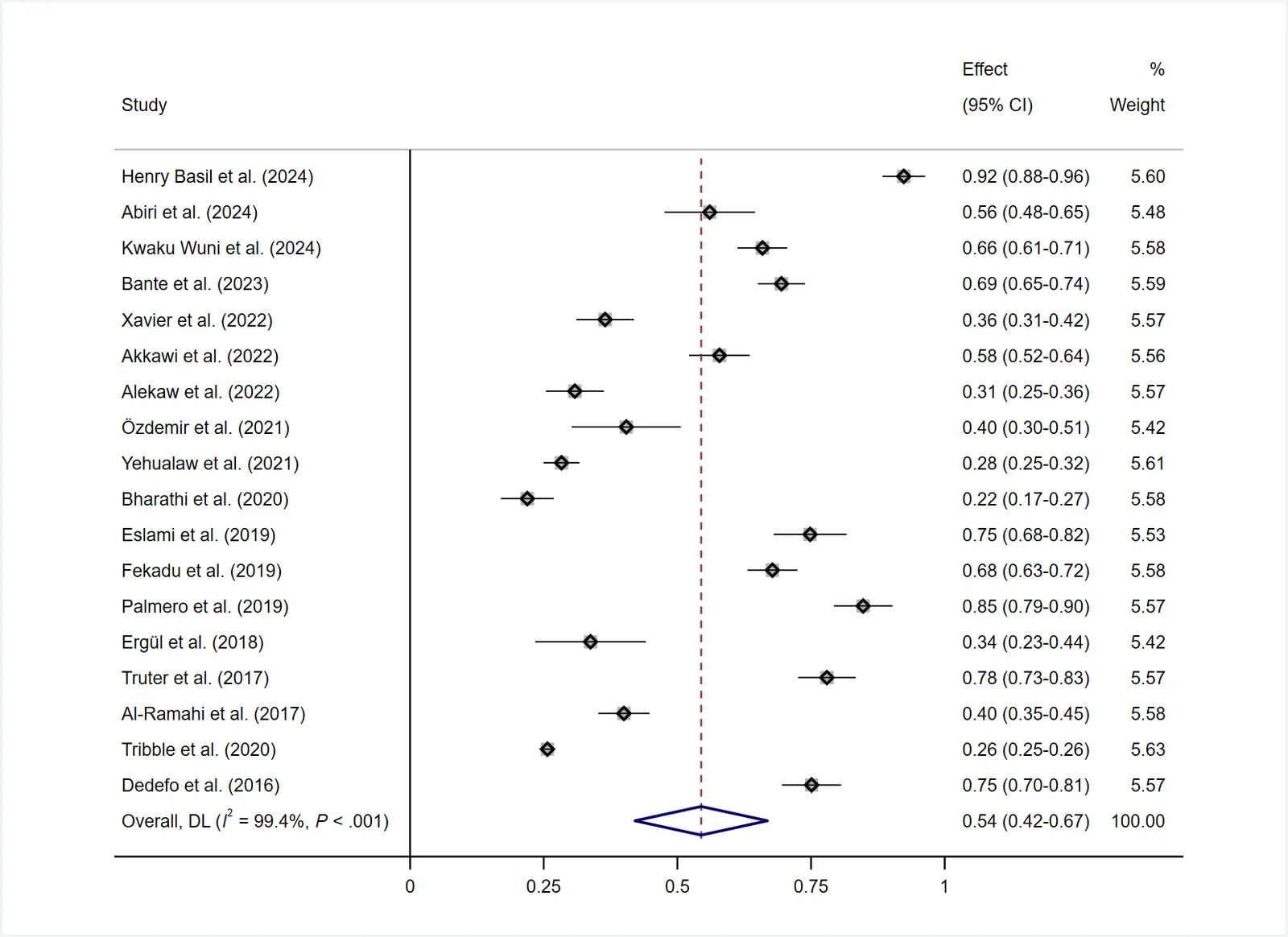
Forest plot of the proportion of hospitalized pediatric patients experiencing medication errors. Weights are from random effects model. DL: DerSimonian-Laird [25,28,29,31-33,36,37,40,41,47-49,52,55,56,61,62].

Based on the denominators used in the included studies, we reported the proportion of patients experiencing medication errors, as well as prescribing and administration error rates. Eighteen studies [25,28,29,31-33,36,37,40,41,47,48,52,55,56,61-63] reported the proportion of patients with medication errors. The meta-analysis showed significant heterogeneity across studies (*I*²=99%; *P<*.001). A sensitivity analysis using the leave-one-out method indicated that no single study had a substantial impact on the pooled results. Therefore, a random-effects model was applied. The analysis estimated that 54% of the hospitalized pediatric patients experienced at least 1 medication error (95% CI 42%‐67%), as shown in Figure 3.

### Subgroup Analysis of the Proportion of Patients Experiencing Medication Errors

The results of the subgroup analysis are presented in Table 3, with the number of studies reported for each subgroup. By study design, similar proportions were observed in cohort studies (57%, 95% CI 24%‐90%) and cross-sectional studies (54%, 95% CI 41%‐67%). By national income level, upper-middle–income countries showed a higher proportion (64%, 95% CI 50%‐77%) than other groups, but the number of studies was small. By region, estimates varied from 26% (95% CI 25%‐26%) to 85% (95% CI 78%‐90%). These results should be interpreted with caution due to limited studies and wide CIs. By prescription type, nonantibiotic prescriptions showed a higher proportion (66%, 95% CI 53%‐79%). By time period, the estimates were similar between 2018 and 2025 (55%, 95% CI 37%‐72%) and 2000‐2017 (52%, 95% CI 34%‐69%).

**Table 3.** Subgroup analysis of the proportion of hospitalized pediatric patients experiencing medication errors.

Subgroup	Studies, n	Events, n	Total, n	Prevalence (95% CI)	*P* value	*I*^2^ (%)
Study design						
Cross-sectional	14	4972	15,401	0.54 (0.41-0.67)	<.001	99.30
Cohort	4	551	1072	0.57 (0.24-0.90)	<.001	99.48
Country income level						
High income	2	3166	11,948	0.27 (0.26-0.28)	NA^a^	NA
Upper-middle income	7	945	1412	0.64 (0.50-0.77)	<.001	97.32
Lower-middle income	2	219	669	0.31 (0.28-0.35)	NA	NA
Low income	7	1193	2444	0.52 (0.36-0.68)	<.001	98.64
Study location						
Europe	1	139	164	0.85 (0.78-0.90)	NA	NA
Asia	6	564	1055	0.54 (0.28-0.80)	<.001	99.07
Africa	9	1633	3070	0.56 (0.43-0.70)	<.001	98.60
North America	1	3027	11,784	0.26 (0.25-0.26)	NA	NA
Special regions	1	160	400	0.40 (0.35-0.45)	NA	NA
Prescription type						
Antibiotic prescriptions	7	3653	13,523	0.36 (0.28-0.44)	<.001	95.93
Nonantibiotic prescriptions	11	1870	2950	0.66 (0.53-0.79)	<.001	98.51
Study period						
2010‐2017	9	4000	13,559	0.52 (0.34-0.69)	<.001	99.33
2018‐2025	8	1346	2687	0.55 (0.37-0.72)	<.001	99.08
NA	1	177	227	0.78 (0.72-0.83)	NA	NA

a NA: not applicable.

### Meta-Analysis of Medication Administration Errors in Hospitalized Pediatric Patients

**Figure 4.**
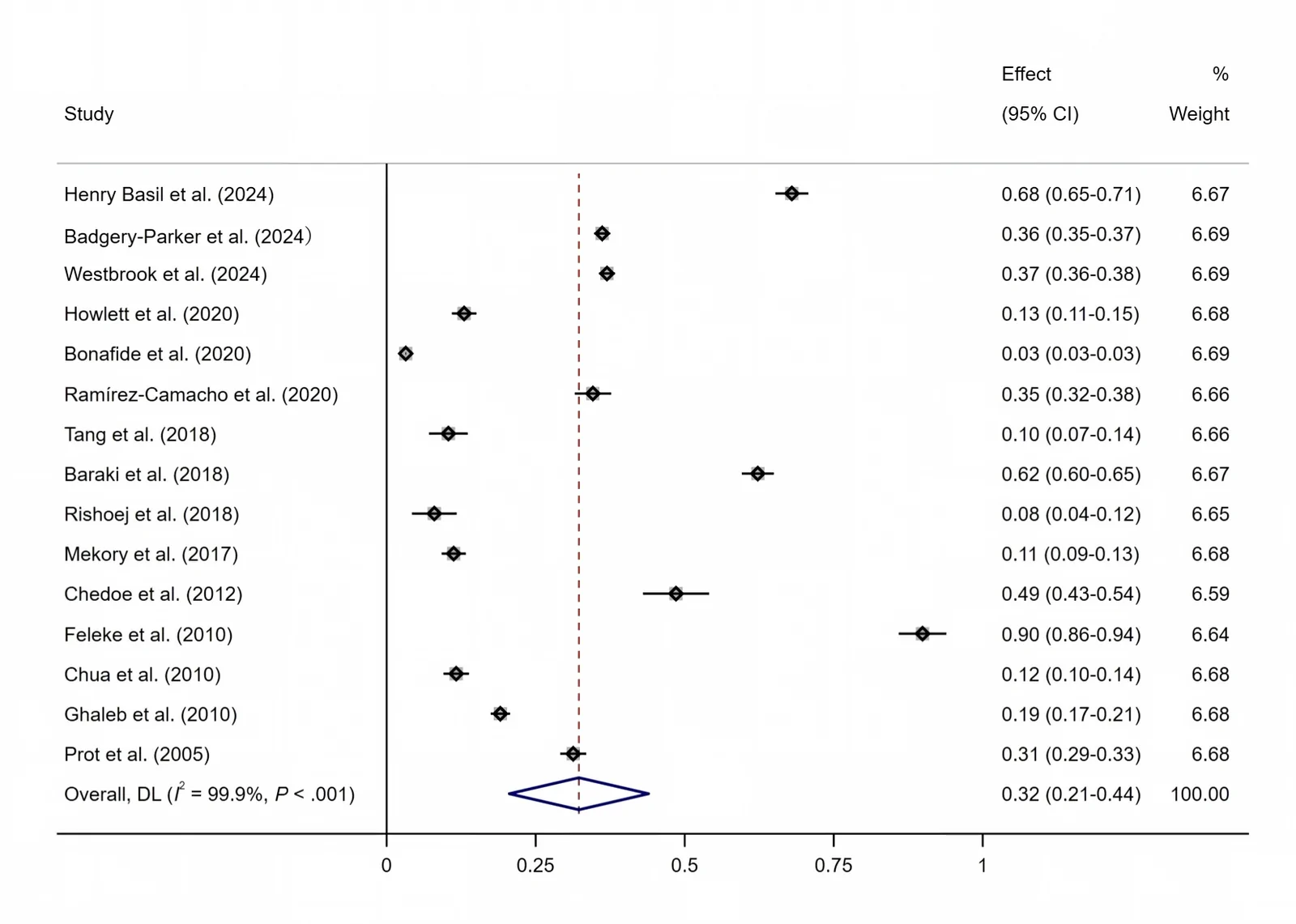
Forest plot of medication administration error rates in hospitalized pediatric patients. Weights are from random effects model. DL: DerSimonian-Laird [25-27,43,45,46,50,53,54,57,68,72-74,82].

Fifteen studies [25-27,43,45,46,50,53,54,57,68,72-74,82] reported medication administration error rates in hospitalized pediatric patients. Meta-analysis revealed substantial heterogeneity among studies (*I*²=99%; *P<*.001). Sensitivity analysis using the leave-one-out method indicated that no single study significantly influenced the overall estimate. Therefore, a random-effects model was applied, showing that the pooled rate of medication administration errors was 32% (95% CI 21%‐44%; Figure 4).

### Subgroup Analysis of Medication Administration Error Rates in Hospitalized Pediatric Patients

The results of the subgroup analysis for administration error rates are presented in Table 4, with the number of studies reported for each subgroup. By study design, cohort studies showed higher estimates (39%, 95% CI 15%‐62%) than cross-sectional studies (29%, 95% CI 16%‐42%). By national income level, low-income countries showed a higher estimate (71%, 95% CI 69%‐73%) compared with other groups, but the number of studies was limited. By region, estimates varied across areas, ranging from 3% (95% CI 3%‐3%) to 71% (95% CI 69%‐73%). These differences should be interpreted cautiously due to limited data and uncertainty. By ward type, ICU (33%, 95% CI 10%‐57%) and general wards (33%, 95% CI 18%‐48%) showed similar estimates. By study period, estimates were 40% (95% CI 20%‐60%) and 29% (95% CI 11%‐47%), with overlapping CIs.

**Table 4.** Subgroup analysis of medication administration error rates in hospitalized pediatric patients.

Subgroup	Studies, n	Events, n	Total, n	Prevalence (95% CI)	*P* value	*I*^2^ (%)
Study design						
Cross-sectional	10	2655	9718	0.29 (0.16-0.42)	<.001	99.64
Cohort	5	12,284	250,218	0.39 (0.15-0.62)	<.001	99.94
Country income level						
High income	9	12,761	255,240	0.23 (0.11-0.35)	<.001	99.87
Upper-middle income	4	1203	3227	0.31 (0.04-0.58)	<.001	99.74
Low income	2	975	1469	0.71 (0.69-0.73)	NA^a^	NA
Study location						
Europe	5	1267	5502	0.24 (0.14-0.33)	<.001	98.61
Asia	4	882	2355	0.25 (−0.00-0.51)	<.001	99.77
Africa	2	975	1469	0.71 (0.69-0.73)	NA	NA
North America	1	7633	238,540	0.03 (0.03-0.03)	NA	NA
Latin America and the Caribbean	1	325	939	0.35 (0.32-0.38)	NA	NA
Australia and New Zealand	2	3757	10,274	0.37 (0.36-0.37)	NA	NA
Ward type						
Intensive care unit	5	8985	241,906	0.33 (0.10-0.57)	<.001	99.86
General ward	6	4192	12,611	0.33 (0.18-0.48)	<.001	99.72
Mixed wards	4	1762	5419	0.30 (0.10-0.50)	<.001	99.65
Study period						
2000‐2012	5	1414	5354	0.40 (0.20-0.60)	<.001	99.69
2013‐2025	7	11,266	248,168	0.29 (0.11-0.47)	<.001	99.91
NA	3	2259	6414	0.27 (0.12-0.42)	NA	NA

a NA: not applicable.

### Meta-Analysis of Factors Associated With Medication Errors in Hospitalized Pediatric Patients

Intravenous administration, hospital stay longer than 5 days, and prescription of 3 or more medications were identified as factors associated with medication errors in hospitalized pediatric patients. Among these, intravenous administration was most strongly associated with medication errors (OR 6.86, 95% CI 3.17‐14.83), followed by the prescription of 3 or more medications (OR 2.12, 95% CI 1.66‐2.70) and a hospital stay longer than 5 days (OR 1.94, 95% CI 1.33‐2.81; Table 5).

**Table 5.** Meta-analysis of factors associated with medication errors in hospitalized pediatric patients.

Associated factors	Number of studies	Minimum OR^a^	Maximum OR	Pooled OR (95% CI)	*I*^2^ (%)	*P* value for heterogeneity
Route of administration (intravenous vs oral)	4	3.36	21.18	6.86 (3.17-14.83）	89.4	<.001
Sex (male vs female)	3	0.82	1.09	1.00 (0.80-1.26）	0.0	.61
Hospital stay (>5 days vs <5 days)	5	1.34	3.46	1.94 (1.33-2.81）	63.1	.03
Number of prescribed medications (≥3 vs 1‐2)	5	1.24	7.02	2.12 (1.66-2.70）	40.1	.15
Weight (>20 kg vs <10 kg)	2	1.13	1.7	1.28 (0.63-2.58)	0.0	.60
Night shift	2	0.81	3.06	1.52 (0.41-5.60)	94.9	<.001

a OR: odds ratio.

### Sensitivity Analysis

Sensitivity analyses were conducted using a leave-one-out approach. Sequential exclusion of each study did not materially change the pooled estimates for prescribing errors, proportion of patients experiencing medication errors, or administration errors, indicating the robustness of the findings (Figures S1-S3 in Multimedia Appendix 3). To assess the robustness of the pooled estimates, the results obtained using the DerSimonian-Laird method were further validated using the REML method. The pooled effect estimates derived from the 2 methods were highly consistent, with substantial overlap in the corresponding 95% CIs, indicating good robustness of the study findings (Figures S4-S6 in Multimedia Appendix 3).

### Publication Bias

Because fewer than 10 studies were included for each factor associated with medication errors, publication bias was only assessed for medication error rates. Multiple methods were used to evaluate publication bias. Funnel plots for prescribing errors, administration errors, and proportion of patients with medication errors showed asymmetry (Figures 7-9 in Multimedia Appendix 3). Egger tests indicated potential publication bias for prescribing errors and administration errors (*z*=9.39; *P*<.001 and *z*=2.25; *P*=.03). However, nonparametric trim-and-fill analyses (imputed studies=0) suggested that no additional studies were needed. Therefore, the observed asymmetry likely reflects true heterogeneity between studies rather than solely publication bias.

## Discussion

### Principal Findings

This systematic review and meta-analysis evaluated the overall medication error rates and associated factors in hospitalized pediatric patients. We found that 54% of the pediatric inpatients experienced a medication error, which is lower than the 69.5% reported by Bante et al [31]. The pooled prescribing error rate was 28%, comparable to the 28.9% reported by Palmero et al [63] but higher than the 17.5% reported by Koumpagioti et al [84]. The administration error rate was 32%, slightly lower than the 37% reported by Westbrook et al [27]. These discrepancies may be attributable to differences in the characteristics of the included populations, definitions of medication errors, and data collection methods. Furthermore, intravenous administration, hospital stay longer than 5 days, and prescription of 3 or more medications were identified as factors associated with medication errors, providing clinically relevant insights for error prevention strategies.

This systematic review and meta-analysis evaluated prescribing error rates, the proportion of patients experiencing medication errors, administration error rates, and associated factors among hospitalized pediatric patients. Notably, substantial heterogeneity was observed across all primary outcomes, and therefore the pooled estimates should be interpreted with caution. The observed heterogeneity may be attributable to methodological and clinical differences among the included studies [85]. Specifically, studies varied in their units of analysis, with some reporting errors per patient and others per prescription or medication administration. Variations were also identified in medication error detection methods, including direct observation, medical record review, and database-based data extraction, which may differ in their ability to identify errors. Furthermore, the included studies were conducted across diverse clinical settings, such as general pediatric wards, NICUs, and PICUs, where patient characteristics, medication-use processes, and risk profiles may differ substantially. In addition, definitions of medication errors were not uniform across studies. Collectively, these factors may have contributed to the high level of heterogeneity and may limit the generalizability of the pooled findings [86]. Therefore, the results of this review should be interpreted as reflecting the overall trends across the included studies rather than precise estimates applicable to all pediatric inpatient settings.

Subgroup analyses across prescription, patient, and administration dimensions highlight the complexity and heterogeneity of pediatric medication errors. Geographic and economic differences were observed, with higher estimates reported in Latin America and the Caribbean, Africa, and low- to middle-income countries. These findings may reflect differences in health care resources, reporting practices, and system development; however, they should be interpreted cautiously given the limited number of studies in several subgroups and substantial heterogeneity. Lower estimates observed in North America may be associated with more developed medication safety systems; however, this explanation remains speculative and was not directly examined in the included studies. Temporal trends showed higher prescribing error rates and a higher proportion of patients experiencing medication errors in more recent studies, which may reflect improved detection and reporting practices over time. In contrast, administration error rates appeared to decrease in recent years. PICUs consistently showed higher error rates, likely reflecting the complexity and high-risk nature of these clinical settings [87,88].

The findings of this study suggest that medication errors in hospitalized pediatric patients are closely associated with treatment-related factors, including intravenous administration, hospital stay longer than 5 days, and the prescription of 3 or more medications. However, as this study is based on observational data, the results reflect statistical associations rather than causal relationships, and residual confounding cannot be excluded.

Intravenous administration was associated with a higher likelihood of medication errors compared with oral administration (OR 6.86). This finding suggests that intravenous delivery may be an important factor associated with medication errors. Similar results have been reported by Westbrook et al [27], who observed 5137 medication administration events in hospitalized children and found that intravenous medications were associated with higher error rates, reaching 77.4% for injections and 64.7% for infusions. The complexity of intravenous medication processes, including dose calculation, dilution, preparation, and infusion rate adjustment, may contribute to the increased risk of errors. Pediatric dosing, which is often based on body weight or body surface area [62], further increases complexity. Rajakumar et al [89] also reported that such individualized dosing increases the risk of medication errors. In addition, pediatric doses are often substantially lower than adult doses, and even minor calculation or measurement errors may result in clinically significant dosing deviations, potentially leading to serious harm [74].

Patients with a hospital stay longer than 5 days were more likely to experience medication errors compared with those with a stay of 5 days or less (OR 1.94). This finding is consistent with the study by Bante et al [31], which also reported that longer hospital stays were associated with a higher risk of medication errors. Longer hospitalization may be associated with increased exposure to medications, resulting in a greater number of prescribing and administration processes, which in turn increases the opportunity for errors. However, any assumption that prolonged hospitalization directly leads to reduced adherence to verification procedures should be interpreted with caution, as this was not directly examined in the included studies.

Children receiving 3 or more prescribed medications had a higher likelihood of medication errors compared with those receiving fewer than 3 medications (OR 2.12). However, evidence regarding the underlying mechanisms of this association remains limited. This finding may be related to increased complexity in medication management as the number of prescriptions increases, including prescribing, verification, dispensing, and administration processes. It may also reflect greater exposure to medication-related procedures in patients receiving multiple drugs. In addition, patients receiving polypharmacy are often clinically more complex, which may contribute to higher observed error rates. Nevertheless, these explanations remain speculative, as the included studies did not provide sufficient information on drug classes, dosing frequency, or disease severity. Therefore, further research is needed to clarify the relationship between polypharmacy and medication errors and to explore potential underlying mechanisms.

### Limitations

This study has several limitations. Although uniform inclusion criteria were applied, the definitions and classification systems for medication errors varied across the included studies. This may have led to misclassification bias, as certain error types may have been included in some studies but excluded in others. In addition, only studies published in English were included, which may have resulted in the omission of relevant evidence published in other languages, potentially limiting the global generalizability of the findings. Substantial heterogeneity was observed across the included studies, which may limit the interpretability and generalizability of the pooled estimates. Furthermore, only 3 outcomes were quantitatively synthesized, including the proportion of patients experiencing medication errors, prescription error rates, and administration error rates. Although some studies reported additional types of medication errors, such as dispensing errors and reconstitution errors, the limited number of studies and inconsistencies in reporting prevented meaningful meta-analysis of these outcomes. Therefore, the findings may not fully capture the overall burden of all types of medication errors in hospitalized pediatric patients. Future studies should adopt more standardized reporting frameworks for different types of medication errors to enable more comprehensive evidence synthesis.

### Conclusions

The findings of this study indicate that medication errors remain a significant patient safety concern among hospitalized pediatric populations worldwide. Prescription errors and administration errors are the most frequently reported and relatively well-evidenced types of medication errors, and a substantial proportion of hospitalized pediatric patients experience at least 1 medication error. Intravenous administration, hospital stay longer than 5 days, and the prescription of 3 or more medications may be associated with an increased risk of medication errors, suggesting that treatment-related factors are closely linked to medication safety in pediatric inpatients. However, these findings should be interpreted as observational associations rather than causal relationships. These results highlight the importance of strengthening medication safety practices in pediatric inpatient settings to improve treatment quality and patient safety. Future research is needed to further investigate less-studied types of medication errors, such as dispensing and reconstitution errors, to provide a more comprehensive understanding of the overall burden and to support the development of targeted interventions.

## Supplementary material

10.2196/100070 Multimedia Appendix 1 The detailed search strategies for each database.

10.2196/100070 Multimedia Appendix 2 Quality assessment of the included cross-sectional and cohort studies

10.2196/100070 Multimedia Appendix 3 Sensitivity analyses, forest plots based on the restricted maximum likelihood random-effects model, and funnel plots for medication error outcomes.

10.2196/100070 Checklist 1 PRISMA 2020 checklist.

## Abbreviations

AHRQ: Agency for Healthcare Research and Quality
NCC MERP: National Coordinating Council for Medication Error Reporting and Prevention
NICU: neonatal intensive care unit
NOS: Newcastle-Ottawa Scale
OR: odds ratio
PICU: pediatric intensive care unit
PRISMA: Preferred Reporting Items for Systematic Reviews and Meta-Analyses
REML: restricted maximum likelihood
