# Supplementary material for: Global Prevalence and Associated Factors of Medication Errors in Hospitalized Pediatric Patients: Systematic Review and Meta-Analysis
Source: JMIR Pediatr Parent. 2026 Aug 27;9:e100070. doi: 10.2196/100070 (PMC13521510; doi:10.2196/100070)
Supplement: Supplementary file 2 [file pediatrics-v9-e100070-s003.docx]

**Multimedia Appendix 3- Figure S1-S9**

Figure S1. Sensitivity Analysis of Studies Reporting Prescription Error Rates

 Figure S2. Sensitivity Analysis of Studies Reporting the Proportion of Patients Experiencing Medication Errors

Figure S3. Sensitivity Analysis of Studies Reporting Medication Administration Error Rates

Figure S4. Forest plot of pooled prescription error rate using REML random-effects model.

Figure S5. Forest plot of pooled proportion of patients experiencing medication errors using REML random-effects model.

Figure S6. Forest plot of pooled medication administration error rate using REML random-effects model.

Figure S7. Funnel Plot of Studies Reporting Prescription Error Rates

Figure S8. Funnel Plot of Studies Reporting the Proportion of Patients Experiencing Medication Errors

Figure S9. Funnel Plot of Studies Reporting Medication Administration Error Rates
