# Supplementary material for: Global Prevalence and Associated Factors of Medication Errors in Hospitalized Pediatric Patients: Systematic Review and Meta-Analysis
Source: JMIR Pediatr Parent. 2026 Aug 27;9:e100070. doi: 10.2196/100070 (PMC13521510; doi:10.2196/100070)
Supplement: Supplementary file 3 [file pediatrics-v9-e100070-s001.doc]

**Multimedia Appendix 1**-The detailed search strategies for each database

| Embase | 1317 |
| --- | --- |
| Web of sciecnce | 3412 |
| Cochrane | 43 |
| Medline | 1092 |
| Pubmed | 1040 |
| Overall | 6904 |

**Embase**

| #10 | #3 AND #6 AND #9 | 1317 |
| --- | --- | --- |
| #9 | #7 OR #8 | 39876 |
| #8 | 'medication error*':ab,ti OR 'drug administration error*':ab,ti OR 'erroneous drug administration':ab,ti OR 'medication administration error*':ab,ti OR 'wrong drug administration':ab,ti OR 'drug dispensing error':ab,ti OR 'medication dispensing error':ab,ti OR 'dos* error':ab,ti OR 'excessive poly*':ab,ti OR 'inappropriate multiple medication':ab,ti OR 'unnecessary polypharmacy':ab,ti OR 'erroneous prescri*':ab,ti OR 'faulty prescri*':ab,ti OR 'inappropriate prescri*':ab,ti OR misprescri*:ab,ti OR 'prescription error*':ab,ti OR 'unsuitable prescri*':ab,ti OR 'wrong prescri*':ab,ti OR 'prescription drug misuse':ab,ti OR 'drug verification error*':ab,ti OR 'transcription error*':ab,ti OR 'transcribing error*':ab,ti | 20108 |
| #7 | 'medication error'/exp OR 'dispensing error'/exp OR 'dosing error'/exp OR 'inappropriate polypharmacy'/exp OR 'prescribing error'/exp | 32814 |
| #6 | #4 OR #5 | 1046499 |
| #5 | 'hospitali?ed patient*':ab,ti OR 'inpatient*':ab,ti OR 'pediatric inpatients':ab,ti OR 'neonatal icu':ab,ti OR 'newborn icu':ab,ti OR nicu:ab,ti OR 'p$ediatric icu':ab,ti OR 'paediatric intensive care unit*':ab,ti OR 'hospitali?ed child':ab,ti OR 'hospitali?ed adolescent':ab,ti OR 'hospitali?ed teenager*':ab,ti OR 'hospitali?ed infant':ab,ti | 393865 |
| #4 | 'hospital patient'/exp OR 'neonatal intensive care unit'/exp OR 'hospitalization'/exp OR 'hospitalized child'/exp OR 'hospitalized adolescent'/exp OR 'hospitalized infant'/exp OR 'pediatric intensive care unit'/exp | 884349 |
| #3 | #1 OR #2 | 6434015 |
| #2 | 'child*':ab,ti OR 'infant*':ab,ti OR 'toddler*':ab,ti OR 'youth':ab,ti OR 'teenager*':ab,ti OR 'p?ediatric*':ab,ti OR 'preschooler*':ab,ti OR 'preschool child':ab,ti OR 'school child*':ab,ti OR 'neonat*':ab,ti OR 'kid*':ab,ti OR 'baby':ab,ti | 4084971 |
| #1 | 'child'/exp OR 'infant'/exp OR 'toddler'/exp OR 'juvenile'/exp OR 'adolescent'/exp OR 'pediatrics'/exp OR 'preschool child'/exp OR 'school child'/exp OR 'newborn'/exp | 4807511 |

**Web of sciecnce**

((TS=('child*' OR 'infant*' OR 'toddler*' OR 'youth' OR 'juvenile' OR 'adolescent' OR 'teenager*' OR 'p?ediatric*' OR 'preschooler*' OR 'preschool child' OR 'school child*' OR 'newborn' OR 'neonat*' OR 'kid*' OR 'baby')) AND TS=('hospitali?ed patient*' OR 'inpatient*' OR 'pediatric inpatients' OR 'neonatal intensive care unit' OR 'neonatal ICU' OR 'newborn ICU' OR 'NICU' OR 'p$ediatric ICU' OR 'p$ediatric intensive care unit*' OR 'hospitalization' OR 'hospitali?ed child' OR 'hospitali?ed adolescent' OR 'hospitali?ed teenager*' OR 'hospitali?ed infant' )) AND TS=('medication error*' OR 'drug administration error*' OR 'erroneous drug administration' OR 'medication administration error*' OR 'wrong drug administration' OR 'dispensing error' OR 'drug dispensing error' OR 'medication dispensing error' OR 'dos* error' OR 'inappropriate polypharmacy' OR 'excessive poly*' OR 'inappropriate multiple medication' OR 'unnecessary polypharmacy' OR 'prescribing error' OR 'erroneous prescri*' OR 'faulty prescri*' OR 'inappropriate prescri*' OR 'misprescri*' OR 'prescription error*' OR 'unsuitable prescri*' OR 'wrong prescri*' OR 'prescription drug misuse' OR 'drug verification error*' OR 'transcription error*' OR 'transcribing error*' )

**Cochrane**

(child OR infant OR toddler OR youth OR juvenile OR adolescent OR teenager OR p?ediatric OR preschooler OR "preschool child" OR "school child" OR newborn OR neonate OR kid OR baby) AND ("hospitalized patient" OR inpatient OR "pediatric inpatient" OR "neonatal intensive care unit" OR "neonatal ICU" OR "newborn ICU" OR NICU OR "pediatric ICU" OR "pediatric intensive care unit" OR hospitalization OR "hospitalized child" OR "hospitalized adolescent" OR "hospitalized teenager" OR "hospitalized infant") AND ("medication error" OR "drug administration error" OR "erroneous drug administration" OR "medication administration error" OR "wrong drug administration" OR "drug dispensing error" OR "medication dispensing error" OR "dosing error" OR "inappropriate polypharmacy" OR "excessive polymedication" OR "excessive polypharmacy" OR "inappropriate multiple medication" OR "unnecessary polypharmacy" OR "prescribing error" OR "erroneous prescribing" OR "erroneous prescription" OR "faulty prescribing" OR "faulty prescription" OR "flawed prescribing" OR "inappropriate prescribing" OR "inappropriate prescription" OR "misprescribing" OR "misprescription" OR "prescribing fault" OR "prescription error" OR "prescription fault" OR "unsuitable prescribing" OR "unsuitable prescription" OR "wrong prescribing" OR "wrong prescription" OR "prescription drug misuse" OR "drug verification error" OR "transcription error" OR "transcribing error" )

**MEDLINE**

| #13 | #4 and #9 and #12 | 1092 |
| --- | --- | --- |
| #12 | #10 or #11 | 32988 |
| #11 | ('medication error*' or 'drug administration error*' or 'erroneous drug administration' or 'medication administration error*' or 'wrong drug administration' or 'dispensing error' or 'drug dispensing error' or 'medication dispensing error' or 'dos* error' or 'inappropriate polypharmacy' or 'excessive poly*' or 'inappropriate multiple medication' or 'unnecessary polypharmacy' or 'prescribing error' or 'erroneous prescri*' or 'faulty prescri*' or 'inappropriate prescri*' or 'misprescri*' or 'prescription error*' or 'unsuitable prescri*' or 'wrong prescri*' or 'prescription drug misuse' or 'drug verification error*' or 'transcription error*' or 'transcribing error*').ab,kw,ti. | 13873 |
| #10 | inappropriate prescribing/ or medication errors/ or polypharmacy/ | 26089 |
| #9 | #5 or #6 or #7 or #8 | 513598 |
| #8 | ('hospitali?ed patient*' or 'inpatient*' or 'pediatric inpatients' or 'neonatal intensive care unit' or 'neonatal ICU' or 'newborn ICU' or 'NICU' or 'p$ediatric ICU' or 'p$ediatric intensive care unit*' or 'hospitalization' or 'hospitali?ed child' or 'hospitali?ed adolescent' or 'hospitali?ed teenager*' or 'hospitali?ed infant').ab,kw,ti. | 407750 |
| #7 | intensive care, neonatal/ or hospitalization/ | 153940 |
| #6 | intensive care units, pediatric/ or intensive care units, neonatal/ | 30499 |
| #5 | adolescent, hospitalized/ or child, hospitalized/ or inpatients/ | 40521 |
| #4 | #1 or #2 or #3 | 5576745 |
| #3 | ('child*' or 'infant*' or 'toddler*' or 'youth' or 'juvenile' or 'teenager*' or 'p?ediatric*' or 'preschooler*' or 'preschool child' or 'school child*' or 'neonat*' or 'kid*' or 'baby').ab,kw,ti. | 3337601 |
| #2 | pediatrics/ or neonatology/ | 62903 |
| #1 | adolescent/ or exp child/ or exp infant/ | 4172793 |

**Pubmed**

| #10 | #3 and #6 and #9 | 1,040 |
| --- | --- | --- |
| #9 | #7 or #8 | 27,885 |
| #8 | "medication error*"[Title/Abstract] OR "drug administration error*"[Title/Abstract] OR "erroneous drug administration"[Title/Abstract] OR "medication administration error*"[Title/Abstract] OR "wrong drug administration"[Title/Abstract] OR "dispensing error"[Title/Abstract] OR "drug dispensing error"[Title/Abstract] OR "medication dispensing error"[Title/Abstract] OR "dosing error"[Title/Abstract] OR "inappropriate polypharmacy"[Title/Abstract] OR "excessive poly*"[Title/Abstract] OR "unnecessary polypharmacy"[Title/Abstract] OR "prescribing error"[Title/Abstract] OR "erroneous prescri*"[Title/Abstract] OR "faulty prescri*"[Title/Abstract] OR "inappropriate prescri*"[Title/Abstract] OR "misprescri*"[Title/Abstract] OR "prescription error*"[Title/Abstract] OR "wrong prescri*"[Title/Abstract] OR "prescription drug misuse"[Title/Abstract] OR "transcription error*"[Title/Abstract] OR "transcribing error*"[Title/Abstract] | 13,361 |
| #7 | "Medication Errors"[Mesh] | 21,286 |
| #6 | #4 or #5 | 649,162 |
| #5 | "hospitali*ed patient*"[Title/Abstract] OR "inpatient*"[Title/Abstract] OR "pediatric inpatients"[Title/Abstract] OR "neonatal intensive care unit"[Title/Abstract] OR "neonatal ICU"[Title/Abstract] OR "newborn ICU"[Title/Abstract] OR "NICU"[Title/Abstract] OR "pediatric ICU"[Title/Abstract] OR "pediatric intensive care unit*"[Title/Abstract] OR "hospitalization"[Title/Abstract] OR "hospitali*ed child"[Title/Abstract] OR "hospitali*ed adolescent"[Title/Abstract] OR "hospitali*ed teenager*"[Title/Abstract] OR "hospitali*ed infant"[Title/Abstract] | 420,351 |
| #4 | ((((("Inpatients"[Mesh]) OR "Intensive Care Units, Neonatal"[Mesh]) OR "Intensive Care Units, Pediatric"[Mesh]) OR "Hospitalization"[Mesh]) OR "Child, Hospitalized"[Mesh]) OR "Adolescent, Hospitalized"[Mesh] | 371,614 |
| #3 | #1 or #2 | 5,057,525 |
| #2 | "child*"[Title/Abstract] OR "infant*"[Title/Abstract] OR "toddler*"[Title/Abstract] OR "youth"[Title/Abstract] OR "juvenile"[Title/Abstract] OR "adolescent"[Title/Abstract] OR "teenager*"[Title/Abstract] OR "pediatric*"[Title/Abstract] OR "preschooler*"[Title/Abstract] OR "preschool child"[Title/Abstract] OR "school child*"[Title/Abstract] OR "newborn"[Title/Abstract] OR "neonat*"[Title/Abstract] OR "kid"[Title/Abstract] OR baby[Title/Abstract] | 2,879,372 |
| #1 | (((((Child[MeSH Terms]) OR (Child, Preschool[MeSH Terms])) OR (Infant[MeSH Terms])) OR (Infant, Newborn[MeSH Terms])) OR (Adolescent[MeSH Terms])) OR (Pediatrics[MeSH Terms]) | 4,184,756 |
