# Supplementary material for: Global Prevalence and Associated Factors of Medication Errors in Hospitalized Pediatric Patients: Systematic Review and Meta-Analysis
Source: JMIR Pediatr Parent. 2026 Aug 27;9:e100070. doi: 10.2196/100070 (PMC13521510; doi:10.2196/100070)
Supplement: Supplementary file 4 [file pediatrics-v9-e100070-s002.doc]

**Multimedia Appendix 2**-Table S1. Quality assessment of the included cross-sectional studies

| **Study** | **①** | **②** | **③** | **④** | **⑤** | **⑥** | **⑦** | **⑧** | **⑨** | **⑩** | **⑪** | **Total score** |
| --- | --- | --- | --- | --- | --- | --- | --- | --- | --- | --- | --- | --- |
| Canales-Siguero et al. (2025) | Yes | Yes | Yes | Yes | No | Yes | Yes | Yes | No | Yes | Unclear | 7 |
| Abiri et al. (2024) | Yes | No | Yes | No | No | Yes | No | Yes | Yes | Yes | Unclear | 6 |
| Kwaku Wuni et al. (2024) | Yes | Yes | Yes | No | Unclear | Yes | Yes | Yes | Unclear | Unclear | No | 6 |
| Bante et al. (2023) | Yes | Yes | Yes | No | No | Yes | No | Yes | No | Yes | Yes | 7 |
| Xavier et al. (2022) | Yes | Yes | Yes | Unclear | Unclear | No | Yes | Yes | No | Yes | Unclear | 6 |
| Akkawi et al. (2022) | Yes | Yes | Yes | Unclear | No | No | Yes | Yes | No | No | No | 5 |
| Garedow et al. (2022) | Yes | Yes | Yes | Unclear | No | Yes | No | Yes | No | Yes | Unclear | 6 |
| Alekaw et al. (2022) | Yes | Yes | Yes | No | Unclear | Yes | No | Yes | Unclear | Yes | Unclear | 6 |
| Özdemir et al. (2021) | Yes | Yes | Yes | Unclear | No | No | Yes | No | Unclear | No | Unclear | 4 |
| Abdel-Qader et al. (2021) | Yes | Yes | Yes | No | No | Yes | No | Yes | Unclear | No | Unclear | 5 |
| Yehualaw et al. (2021) | Yes | Yes | Yes | No | Unclear | Yes | No | Yes | Unclear | Yes | Unclear | 6 |
| Brennan-Bourdon et al. (2020) | Yes | Yes | Yes | Unclear | No | Yes | Yes | No | Unclear | Yes | Unclear | 6 |
| Howlett et al. (2020) | Yes | Yes | Yes | No | No | Yes | Yes | No | No | Yes | Unclear | 6 |
| McMullan et al. (2020) | Yes | Yes | Yes | Unclear | No | Yes | No | Yes | No | Yes | Unclear | 6 |
| Ramírez-Camacho et al. (2020) | Yes | No | Yes | Unclear | Yes | Yes | No | Yes | No | No | Unclear | 5 |
| Eslami et al. (2019) | Yes | Yes | Yes | Unclear | No | No | No | Yes | No | Unclear | Unclear | 4 |
| Fekadu et al. (2019) | Yes | Yes | Yes | Unclear | No | Yes | No | Yes | No | No | Unclear | 5 |
| Palmero et al. (2019) | Yes | Yes | Yes | Unclear | No | Yes | No | Yes | No | Unclear | Unclear | 5 |
| Tang et al. (2018) | Yes | No | Yes | Unclear | No | Yes | No | Yes | No | Yes | Unclear | 5 |
| Kadam et al. (2018) | Yes | No | Yes | Unclear | Yes | No | No | No | Unclear | Yes | Unclear | 4 |
| Ergül et al. (2018) | Yes | Yes | Yes | Unclear | No | No | Yes | No | Unclear | Yes | Unclear | 5 |
| Tribble et al. (2020) | Yes | No | Yes | Unclear | No | Yes | No | Yes | No | Yes | Unclear | 5 |
| Machado et al. (2015) | Yes | No | Yes | Unclear | No | No | Yes | Yes | Yes | Yes | Unclear | 6 |
| Baraki et al. (2018) | Yes | No | Yes | Unclear | Yes | Yes | No | Yes | No | Yes | Unclear | 6 |
| Rishoej et al. (2018) | Yes | No | Yes | Unclear | Yes | Yes | No | No | No | No | No | 4 |
| Truter et al. (2017) | Yes | No | Yes | Unclear | No | Yes | No | No | No | Yes | Unclear | 4 |
| Mekory et al. (2017) | Yes | No | Yes | Unclear | Yes | Yes | Yes | No | No | No | Unclear | 5 |
| Nikhithasri et al. (2017) | Yes | Yes | Yes | No | No | No | Yes | No | No | No | Unclear | 4 |
| Khoo et al. (2017) | Yes | Yes | Yes | No | No | Yes | No | No | No | No | Unclear | 4 |
| Feleke et al. (2010) | Yes | No | Yes | Unclear | No | Yes | No | No | Unclear | Yes | Unclear | 4 |
| Chua et al. (2010) | Yes | No | Yes | Unclear | No | Yes | No | Yes | No | Yes | Unclear | 5 |
| Ghaleb et al. (2010) | Yes | No | Yes | Unclear | No | Yes | Yes | No | Unclear | Yes | Unclear | 5 |
| Otero et al. (2008) | Yes | No | Yes | Unclear | No | No | No | Yes | No | Yes | Unclear | 4 |
| Wang et al. (2007) | Yes | Yes | Yes | Yes | Yes | Yes | Yes | No | No | No | Unclear | 7 |
| Prot et al. (2005) | Yes | No | Yes | Yes | No | Yes | No | Yes | No | No | Unclear | 5 |

①Define the source of information (survey, record review)	②List inclusion and exclusion criteria for exposed and unexposed subjects (cases and controls) or refer to previous publications	③Indicate time period used for identifying patients	④Indicate whether or not subjects were consecutive if not population-based

⑤Indicate if evaluators of subjective components of study were masked to other aspects of the status of the participants	⑥Describe any assessments undertaken for quality assurance purposes (e.g., test/retest of primary outcome measurements)	⑦Explain any patient exclusions from analysis	⑧Describe how confounding was assessed and/or controlled.	⑨If applicable, explain how missing data were handled in the analysis	⑩ Summarize patient response rates and completeness of data collection	⑪Clarify what follow-up, if any, was expected and the percentage of patients for which incomplete data or follow-up was obtained

| **Multimedia Appendix 2**-Table S2. Quality assessment of the included cohort studies | | | | | | | | | |
| --- | --- | --- | --- | --- | --- | --- | --- | --- | --- |
| **Study** | **Representativeness of the exposed cohort** | **Selection of the non exposed cohort** | **Ascertainment of exposure** | **Demonstration that outcome of interest was not present at start of study** | **Comparability of cohorts on the basis of the design or analysis** | **Assessment of outcome** | **Was follow-up long enough for outcomes to occur** | **Adequacy of follow up of cohorts** | **Total score** |
| Henry Basil et al. (2025) | 1 | 0 | 1 | 1 | 2 | 1 | 1 | 1 | 8 |
| Badgery-Parker et al. (2024) | 1 | 0 | 1 | 1 | 2 | 1 | 1 | 0 | 7 |
| Westbrook et al. (2024) | 1 | 0 | 1 | 1 | 2 | 1 | 1 | 1 | 8 |
| Satir et al. (2023) | 1 | 1 | 1 | 0 | 1 | 1 | 0 | 1 | 6 |
| Nasso et al. (2022) | 1 | 0 | 1 | 0 | 2 | 1 | 1 | 1 | 7 |
| Kadmon et al. (2021) | 1 | 0 | 1 | 1 | 2 | 1 | 1 | 0 | 7 |
| Bharathi et al. (2020) | 1 | 0 | 1 | 1 | 2 | 0 | 1 | 0 | 6 |
| Bonafide et al. (2020) | 1 | 1 | 1 | 1 | 2 | 1 | 1 | 0 | 8 |
| Al-Ramahi et al. (2017) | 1 | 0 | 1 | 1 | 2 | 0 | 0 | 1 | 6 |
| Stultz et al. (2014) | 1 | 0 | 1 | 1 | 2 | 0 | 1 | 1 | 7 |
| Kunac et al. (2010) | 1 | 1 | 1 | 1 | 2 | 1 | 1 | 0 | 8 |
| Ligi et al. (2008) | 1 | 1 | 1 | 1 | 2 | 1 | 1 | 0 | 8 |
| Walsh et al. (2006) | 1 | 0 | 1 | 1 | 1 | 1 | 1 | 1 | 7 |
| Ewig et al. (2017) | 1 | 0 | 1 | 1 | 1 | 1 | 1 | 1 | 7 |
| Dedefo et al. (2016) | 1 | 0 | 1 | 1 | 2 | 1 | 0 | 0 | 6 |
| Palmero et al. (2016) | 1 | 1 | 1 | 1 | 2 | 1 | 1 | 0 | 8 |
| Glanzmann et al. (2015) | 1 | 1 | 1 | 1 | 2 | 1 | 1 | 0 | 8 |
| Sakuma et al. (2014) | 1 | 1 | 1 | 1 | 2 | 1 | 1 | 1 | 9 |
| Chedoe et al. (2012) | 1 | 1 | 1 | 1 | 2 | 1 | 0 | 1 | 8 |
| Booth et al. (2012) | 1 | 1 | 1 | 1 | 2 | 1 | 1 | 0 | 8 |
| Al-Jeraisy et al. (2011) | 1 | 0 | 1 | 1 | 0 | 1 | 0 | 1 | 5 |
| Campino et al. (2008) | 1 | 1 | 1 | 1 | 2 | 1 | 0 | 0 | 7 |
| Parihar et al. (2008) | 1 | 0 | 1 | 1 | 1 | 0 | 1 | 0 | 5 |
| Buckley et al. (2007) | 1 | 0 | 1 | 1 | 1 | 1 | 1 | 0 | 6 |
| Potts et al. (2004) | 1 | 1 | 1 | 1 | 2 | 1 | 1 | 0 | 8 |
| Kaushal et al. (2001) | 1 | 0 | 1 | 1 | 2 | 1 | 1 | 1 | 8 |
